# (*E*)-3-(1,3-Diphenyl-1*H*-pyrazol-4-yl)-1-(thia­zol-2-yl)prop-2-en-1-one

**DOI:** 10.1107/S2414314623009975

**Published:** 2023-11-21

**Authors:** Dongsoo Koh

**Affiliations:** a Department of Applied Chemistry, Dongduk Women’s University, Seoul 136-714, Republic of Korea; University of Aberdeen, United Kingdom

**Keywords:** crystal structure, chalcone, heterocycles, C—H⋯O hydrogen bonds

## Abstract

In the title mol­ecule, C_21_H_15_N_3_OS, the C5=C6 double bond in the central enone group adopts a *trans* configuration. The dihedral angle between planes of the thia­zole and pyrazole rings is 6.6 (2)°. In the crystal, pairs of C—H⋯O hydrogen bonds generate inversion dimers and another pair of C—H⋯N hydrogen bonds link the dimers into chains propagating along *a*-axis direction.

## Abstract 2

The crystal structure of a heterocycles containing chalcone is reported.

## Graphical abstract

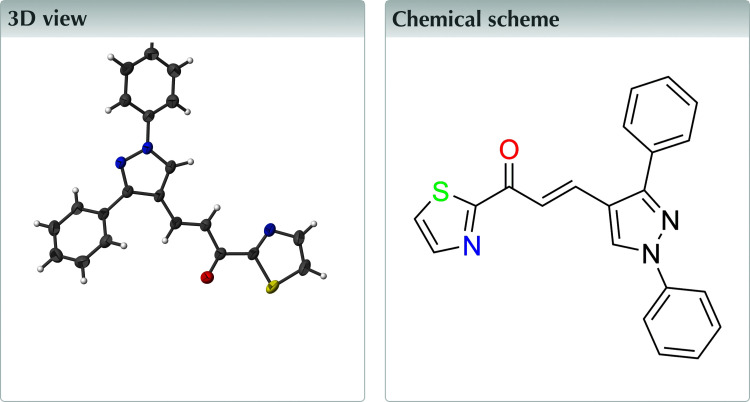

## Structure description

Chalcones commonly contain a C6—C3—C6 skeleton, of which C3 represents an α,β-unsaturated carbonyl (enone) group, and the two C6s represent phenyl groups attached to both ends of the enone group. Chalcones, which are secondary metabolites of plants, have been shown to possess diverse biological activities including anti­cancer (Ouyang *et al.*, 2021 ▸), anti-diabetic (Welday Kahssay *et al.*, 2021 ▸), anti-microbial (Henry *et al.* 2020 ▸), and anti­viral (Fu *et al.*, 2020 ▸). According to recent studies, heterocycles exhibit better physiological activity than phenyl groups, so research is actively underway to replace the phenyl groups of chalcone with heterocycles (Elkanzi *et al.*, 2022 ▸). As a continuation of our research program in this area (Jeong *et al.*, 2020 ▸; Shin *et al.*, 2020 ▸), the title chalcone containing a heterocycle was designed and synthesized.

**Figure 1:**
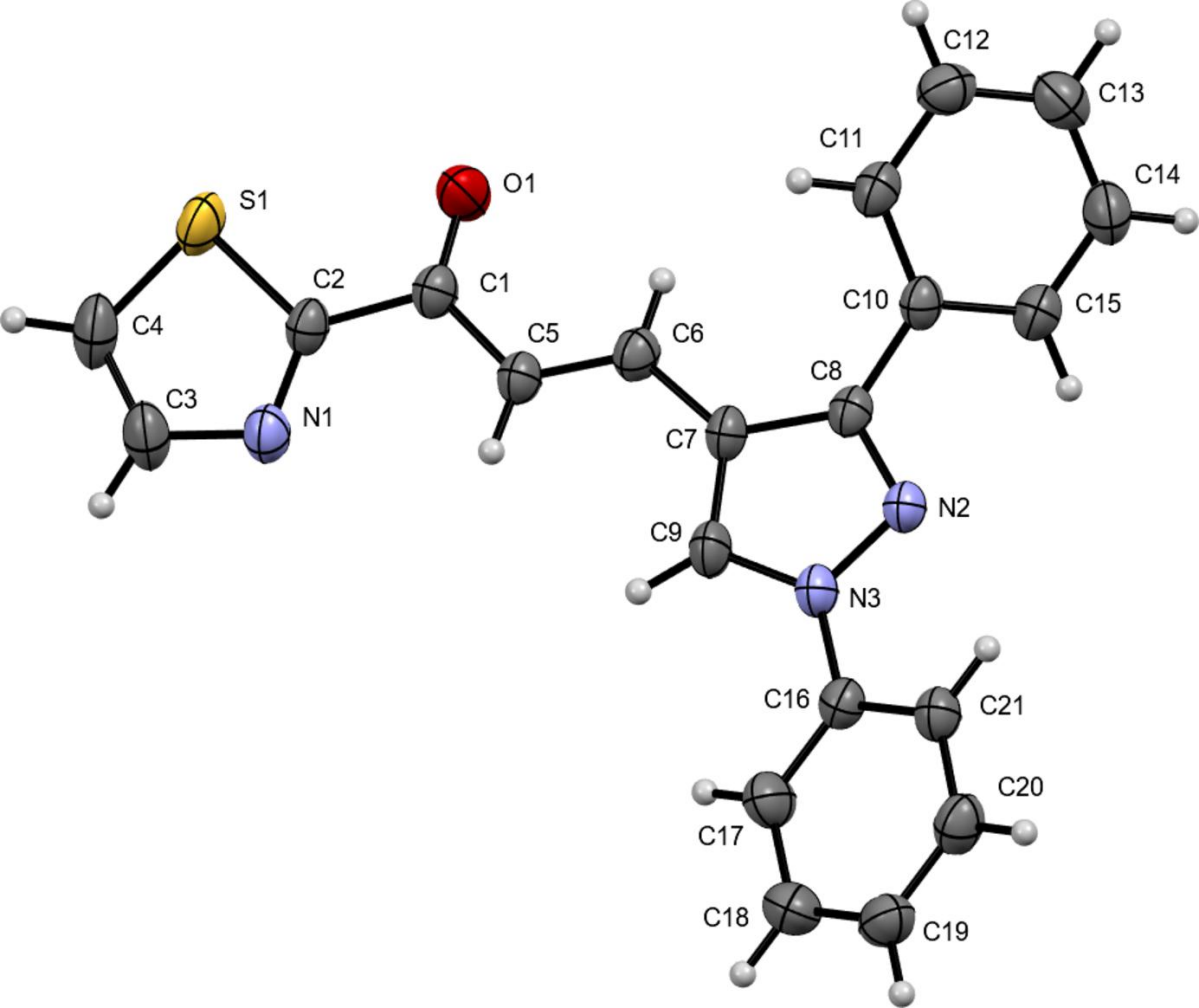
The mol­ecular structure of the title compound, showing displacement ellipsoids drawn at the 30% probability level.

The mol­ecular structure of the title compound is shown in Fig. 1 ▸. The *trans* configuration of the C5=C6 double bond in the central enone group is confirmed by the dihedral angle of C1—C5=C6—C7 of 179.25 (19)°. The title chalcone mol­ecule has a thia­zole ring and a pyrazole ring attached to both sides of the enone group. The dihedral angle between the thia­zole ring (C2/N1/C3/C4/S1) and the pyrazole ring (C7/C8/N2/N3/C9) is 6.6 (2)°, indicating that the two rings are almost in the same plane. The pyrazole ring (C7/C8/N2/ N3/C9) has C10–C15 and C16–C21 phenyl groups attached to atoms C8 and N3, respectively. The C10–C15 and C16–C21 phenyl rings make dihedral angles with the pyrazole ring of 38.6 (1) and 25.0 (2)°, respectively, and the dihedral angle between the phenyl rings is 59.9 (3)°.

**Figure 2:**
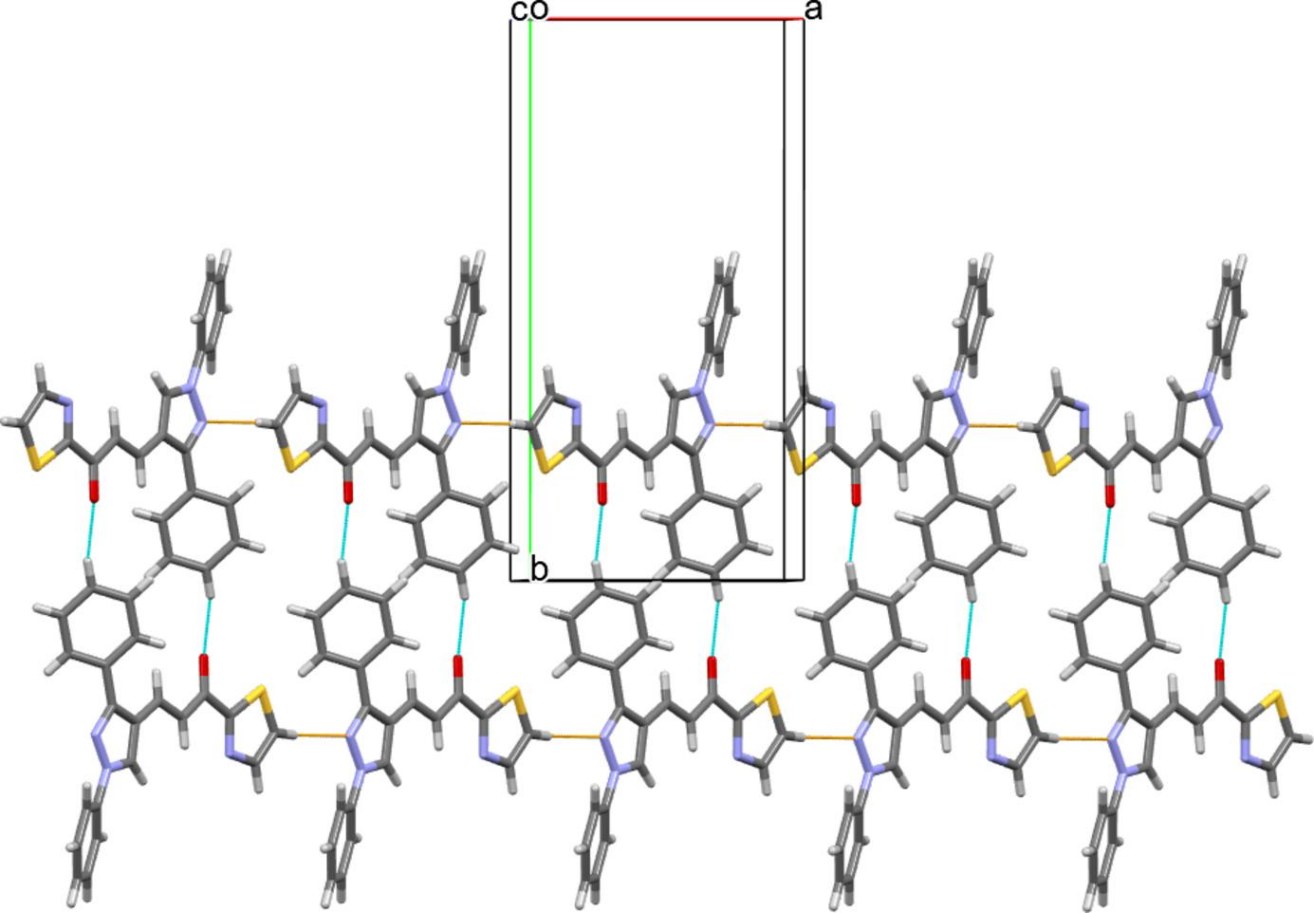
Part of the crystal structure of the title compound, showing the weak C—H⋯O hydrogen bonds forming 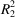 (22) dimers as blue lines. An additional pair of inter­molecular hydrogen C—H⋯N bonds (yellow lines) link the dimers into a chain.

In the crystal, pairs of C—H⋯O hydrogen bonds generate inversion dimers with graph-set notation 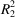 (22) and another pair of C—H⋯N hydrogen bonds link the dimers into chains propagating along [100] (Table 1 ▸, Fig. 2 ▸).

## Synthesis and crystallization

1,3-Diphenyl-1*H*-pyrazole-4-carbaldehyde (248 mg, 1 mmol) and 1-(thia­zol-2-yl)ethanone (127 mg, 1 mmol) were dissolved in 20 ml of ethanol, then the temperature was set to to 276–277 K using an ice bath. To the cooled reaction mixture was added 1.0 ml of 40% aqueous KOH solution, and the reaction mixture was stirred at room temperature for 20 h.

This mixture was poured into iced water (50 ml) and was acidified (pH = 3) with 3 *N* HCl solution to give a precipitate. Filtration and washing with water afforded the crude solid of the title compound (232 mg, 65%). Recrystallization of the solid from ethanol solution gave crystals which were suitable for X-ray diffraction.

## Refinement

Crystal data, data collection and structure refinement details are summarized in Table 2 ▸.

## Supplementary Material

Crystal structure: contains datablock(s) I. DOI: 10.1107/S2414314623009975/hb4458sup1.cif

Structure factors: contains datablock(s) I. DOI: 10.1107/S2414314623009975/hb4458Isup2.hkl

Supporting information file. DOI: 10.1107/S2414314623009975/hb4458Isup3.cml

CCDC reference: 2308467

Additional supporting information:  crystallographic information; 3D view; checkCIF report

## Figures and Tables

**Table 1:** Hydrogen-bond geometry (Å, °)

*D*—H⋯*A*	*D*—H	H⋯*A*	*D*⋯*A*	*D*—H⋯*A*
C18—H18⋯O1^i^	0.94	2.56	3.451 (3)	158
C4—H4⋯N2^ii^	0.94	2.54	3.473 (3)	172
C13—H13⋯O1^iii^	0.94	2.41	3.321 (3)	162

Symmetry codes: (i) 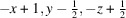 ; (ii) 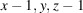 ; (iii) 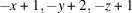 .

**Table 2:** Experimental details

Crystal data	
Chemical formula	C_21_H_15_N_3_OS
*M*_r_	357.42
Crystal system, space group	Monoclinic, *P*2_1_/*c*
Temperature (K)	223
*a*, *b*, *c* (Å)	9.3312 (19), 19.124 (4), 9.977 (2)
β (°)	95.453 (7)
*V* (Å^3^)	1772.4 (6)
*Z*	4
Radiation type	Mo *K*α
μ (mm^−1^)	0.20
Crystal size (mm)	0.14 × 0.14 × 0.06

Data collection	
Diffractometer	Bruker PHOTON III M14
Absorption correction	Multi-scan (*SADABS*; Krause *et al.*, 2015 ▸)
*T*_min_, *T*_max_	0.673, 0.746
No. of measured, independent and observed [*I* > 2σ(*I*)] reflections	39547, 4419, 2775
*R*_int_	0.087
(sin θ/λ)_max_ (Å^−1^)	0.668

Refinement	
*R*[*F* ^2^ > 2σ(*F* ^2^)], *wR*(*F* ^2^), *S*	0.046, 0.127, 1.02
No. of reflections	4419
No. of parameters	235
H-atom treatment	H-atom parameters constrained
Δρ_max_, Δρ_min_ (e Å^−3^)	0.23, −0.26

Computer programs: *APEX2* and *SAINT* (Bruker, 2012 ▸), *SHELXS* and *SHELXTL* (Sheldrick, 2008 ▸), *SHELXL2014* (Sheldrick, 2015 ▸) and *publCIF* (Westrip, 2010 ▸).

## full crystallographic data

### Crystal data

**Table:**

C_21_H_15_N_3_OS	*F*(000) = 744
*M**_r_* = 357.42	*D*_x_ = 1.339 Mg m^−^^3^
Monoclinic, *P*2_1_/*c*	Mo *K*α radiation, λ = 0.71073 Å
*a* = 9.3312 (19) Å	Cell parameters from 5689 reflections
*b* = 19.124 (4) Å	θ = 2.2–27.4°
*c* = 9.977 (2) Å	µ = 0.20 mm^−^^1^
β = 95.453 (7)°	*T* = 223 K
*V* = 1772.4 (6) Å^3^	Block, colourless
*Z* = 4	0.14 × 0.14 × 0.06 mm

### Data collection

**Table:**

Bruker PHOTON III M14 diffractometer	*R*_int_ = 0.087
φ and ω scans	θ_max_ = 28.3°, θ_min_ = 2.2°
Absorption correction: multi-scan (SADABS; Krause *et al.*, 2015)	*h* = −12→12
*T*_min_ = 0.673, *T*_max_ = 0.746	*k* = −25→25
39547 measured reflections	*l* = −13→13
4419 independent reflections	1 standard reflections every 1 reflections
2775 reflections with *I* > 2σ(*I*)	intensity decay: 1%

### Refinement

**Table:**

Refinement on *F*^2^	Primary atom site location: structure-invariant direct methods
Least-squares matrix: full	Hydrogen site location: inferred from neighbouring sites
*R*[*F*^2^ > 2σ(*F*^2^)] = 0.046	H-atom parameters constrained
*wR*(*F*^2^) = 0.127	*w* = 1/[σ^2^(*F*_o_^2^) + (0.0454*P*)^2^ + 0.6998*P*] where *P* = (*F*_o_^2^ + 2*F*_c_^2^)/3
*S* = 1.02	(Δ/σ)_max_ < 0.001
4419 reflections	Δρ_max_ = 0.23 e Å^−^^3^
235 parameters	Δρ_min_ = −0.26 e Å^−^^3^
0 restraints	

### Special details

**Table:**

Geometry. All esds (except the esd in the dihedral angle between two l.s. planes) are estimated using the full covariance matrix. The cell esds are taken into account individually in the estimation of esds in distances, angles and torsion angles; correlations between esds in cell parameters are only used when they are defined by crystal symmetry. An approximate (isotropic) treatment of cell esds is used for estimating esds involving l.s. planes.

### Fractional atomic coordinates and isotropic or equivalent isotropic displacement parameters (Å^2^)

**Table:**

	*x*	*y*	*z*	*U*_iso_*/*U*_eq_	
C1	0.2731 (2)	0.79486 (10)	0.16701 (19)	0.0354 (5)	
C2	0.1738 (2)	0.75669 (10)	0.06690 (19)	0.0341 (4)	
C3	0.0728 (2)	0.67220 (12)	−0.0560 (2)	0.0479 (6)	
H3	0.0573	0.6258	−0.0852	0.058*	
C4	−0.0034 (2)	0.72640 (12)	−0.1141 (2)	0.0507 (6)	
H4	−0.0760	0.7223	−0.1858	0.061*	
C5	0.3616 (2)	0.75244 (11)	0.26503 (19)	0.0344 (4)	
H5	0.3527	0.7035	0.2626	0.041*	
C6	0.4552 (2)	0.78221 (11)	0.35826 (19)	0.0351 (5)	
H6	0.4625	0.8312	0.3574	0.042*	
C7	0.5459 (2)	0.74495 (10)	0.45999 (19)	0.0334 (4)	
C8	0.6387 (2)	0.77245 (10)	0.56977 (18)	0.0310 (4)	
C9	0.5630 (2)	0.67337 (11)	0.4714 (2)	0.0381 (5)	
H9	0.5168	0.6396	0.4141	0.046*	
C10	0.6660 (2)	0.84560 (10)	0.61250 (19)	0.0319 (4)	
C11	0.5555 (2)	0.89512 (11)	0.6078 (2)	0.0377 (5)	
H11	0.4613	0.8823	0.5754	0.045*	
C12	0.5843 (2)	0.96321 (11)	0.6506 (2)	0.0439 (5)	
H12	0.5095	0.9963	0.6462	0.053*	
C13	0.7219 (3)	0.98278 (11)	0.6996 (2)	0.0469 (6)	
H13	0.7408	1.0290	0.7281	0.056*	
C14	0.8320 (2)	0.93384 (12)	0.7065 (2)	0.0469 (5)	
H14	0.9256	0.9466	0.7410	0.056*	
C15	0.8040 (2)	0.86618 (11)	0.6625 (2)	0.0390 (5)	
H15	0.8795	0.8335	0.6665	0.047*	
C16	0.7007 (2)	0.59418 (10)	0.6352 (2)	0.0343 (4)	
C17	0.6949 (2)	0.53556 (11)	0.5536 (2)	0.0469 (6)	
H17	0.6646	0.5396	0.4614	0.056*	
C18	0.7338 (3)	0.47096 (11)	0.6082 (2)	0.0508 (6)	
H18	0.7297	0.4311	0.5528	0.061*	
C19	0.7785 (2)	0.46486 (11)	0.7432 (2)	0.0460 (5)	
H19	0.8041	0.4209	0.7804	0.055*	
C20	0.7852 (2)	0.52363 (11)	0.8236 (2)	0.0430 (5)	
H20	0.8169	0.5195	0.9155	0.052*	
C21	0.7461 (2)	0.58883 (10)	0.7711 (2)	0.0368 (5)	
H21	0.7502	0.6286	0.8268	0.044*	
N1	0.17339 (18)	0.68897 (9)	0.04742 (17)	0.0402 (4)	
N2	0.70582 (17)	0.72116 (8)	0.64143 (16)	0.0331 (4)	
N3	0.65750 (17)	0.66057 (8)	0.57930 (16)	0.0346 (4)	
O1	0.27593 (18)	0.85888 (7)	0.16369 (15)	0.0524 (4)	
S1	0.05109 (6)	0.80275 (3)	−0.03886 (6)	0.04908 (19)	

### Atomic displacement parameters (Å^2^)

**Table:**

	*U*^11^	*U*^22^	*U*^33^	*U*^12^	*U*^13^	*U*^23^
C1	0.0378 (11)	0.0392 (12)	0.0279 (10)	0.0014 (9)	−0.0039 (9)	0.0013 (8)
C2	0.0328 (10)	0.0415 (11)	0.0265 (10)	−0.0015 (8)	−0.0045 (8)	0.0049 (8)
C3	0.0501 (13)	0.0512 (13)	0.0395 (13)	−0.0160 (11)	−0.0110 (10)	0.0025 (10)
C4	0.0437 (13)	0.0643 (15)	0.0406 (13)	−0.0163 (11)	−0.0145 (10)	0.0100 (11)
C5	0.0363 (10)	0.0367 (11)	0.0287 (10)	0.0006 (8)	−0.0046 (8)	−0.0002 (8)
C6	0.0366 (11)	0.0390 (11)	0.0287 (10)	0.0005 (8)	−0.0028 (9)	0.0028 (8)
C7	0.0315 (10)	0.0399 (11)	0.0273 (10)	−0.0008 (8)	−0.0053 (8)	0.0000 (8)
C8	0.0284 (9)	0.0375 (11)	0.0265 (10)	0.0008 (8)	−0.0006 (8)	0.0003 (8)
C9	0.0387 (11)	0.0428 (12)	0.0304 (11)	−0.0017 (9)	−0.0091 (9)	−0.0023 (9)
C10	0.0329 (10)	0.0375 (11)	0.0243 (9)	−0.0009 (8)	−0.0031 (8)	0.0022 (8)
C11	0.0333 (11)	0.0452 (12)	0.0332 (11)	0.0022 (9)	−0.0037 (9)	0.0018 (9)
C12	0.0494 (13)	0.0415 (12)	0.0405 (12)	0.0103 (10)	0.0022 (10)	0.0027 (10)
C13	0.0605 (15)	0.0383 (12)	0.0414 (13)	−0.0072 (10)	0.0023 (11)	−0.0039 (10)
C14	0.0422 (12)	0.0509 (13)	0.0455 (13)	−0.0087 (10)	−0.0063 (10)	−0.0054 (11)
C15	0.0339 (11)	0.0428 (12)	0.0387 (12)	0.0018 (9)	−0.0054 (9)	0.0006 (9)
C16	0.0319 (10)	0.0370 (11)	0.0329 (11)	0.0023 (8)	−0.0027 (8)	0.0000 (8)
C17	0.0585 (14)	0.0443 (13)	0.0355 (12)	0.0063 (11)	−0.0076 (11)	−0.0071 (10)
C18	0.0562 (14)	0.0394 (12)	0.0549 (15)	0.0064 (11)	−0.0044 (12)	−0.0097 (11)
C19	0.0418 (12)	0.0362 (12)	0.0584 (15)	0.0019 (9)	−0.0039 (11)	0.0052 (10)
C20	0.0422 (12)	0.0475 (13)	0.0375 (12)	−0.0017 (10)	−0.0058 (10)	0.0091 (10)
C21	0.0383 (11)	0.0391 (11)	0.0317 (11)	−0.0005 (9)	−0.0034 (9)	−0.0016 (9)
N1	0.0417 (10)	0.0415 (10)	0.0350 (10)	−0.0047 (8)	−0.0079 (8)	0.0026 (8)
N2	0.0339 (9)	0.0344 (9)	0.0294 (9)	0.0003 (7)	−0.0051 (7)	−0.0020 (7)
N3	0.0372 (9)	0.0365 (9)	0.0284 (9)	0.0006 (7)	−0.0064 (7)	−0.0031 (7)
O1	0.0707 (11)	0.0368 (9)	0.0453 (9)	0.0022 (7)	−0.0176 (8)	0.0020 (7)
S1	0.0457 (3)	0.0520 (4)	0.0454 (3)	0.0003 (3)	−0.0178 (3)	0.0110 (3)

### Geometric parameters (Å, º)

**Table:**

C1—O1	1.225 (2)	C11—C12	1.389 (3)
C1—C5	1.464 (3)	C11—H11	0.9400
C1—C2	1.488 (3)	C12—C13	1.381 (3)
C2—N1	1.310 (3)	C12—H12	0.9400
C2—S1	1.7235 (19)	C13—C14	1.387 (3)
C3—C4	1.356 (3)	C13—H13	0.9400
C3—N1	1.366 (3)	C14—C15	1.383 (3)
C3—H3	0.9400	C14—H14	0.9400
C4—S1	1.698 (2)	C15—H15	0.9400
C4—H4	0.9400	C16—C17	1.383 (3)
C5—C6	1.341 (3)	C16—C21	1.386 (3)
C5—H5	0.9400	C16—N3	1.429 (2)
C6—C7	1.446 (3)	C17—C18	1.384 (3)
C6—H6	0.9400	C17—H17	0.9400
C7—C9	1.382 (3)	C18—C19	1.377 (3)
C7—C8	1.430 (2)	C18—H18	0.9400
C8—N2	1.334 (2)	C19—C20	1.379 (3)
C8—C10	1.478 (3)	C19—H19	0.9400
C9—N3	1.348 (2)	C20—C21	1.388 (3)
C9—H9	0.9400	C20—H20	0.9400
C10—C15	1.393 (3)	C21—H21	0.9400
C10—C11	1.397 (3)	N2—N3	1.370 (2)
			
O1—C1—C5	124.01 (18)	C11—C12—H12	119.7
O1—C1—C2	119.07 (17)	C12—C13—C14	119.6 (2)
C5—C1—C2	116.91 (17)	C12—C13—H13	120.2
N1—C2—C1	125.29 (17)	C14—C13—H13	120.2
N1—C2—S1	114.98 (14)	C15—C14—C13	119.9 (2)
C1—C2—S1	119.70 (15)	C15—C14—H14	120.0
C4—C3—N1	116.0 (2)	C13—C14—H14	120.0
C4—C3—H3	122.0	C14—C15—C10	121.2 (2)
N1—C3—H3	122.0	C14—C15—H15	119.4
C3—C4—S1	110.21 (17)	C10—C15—H15	119.4
C3—C4—H4	124.9	C17—C16—C21	120.48 (19)
S1—C4—H4	124.9	C17—C16—N3	119.78 (18)
C6—C5—C1	121.15 (19)	C21—C16—N3	119.73 (17)
C6—C5—H5	119.4	C16—C17—C18	119.9 (2)
C1—C5—H5	119.4	C16—C17—H17	120.1
C5—C6—C7	125.23 (19)	C18—C17—H17	120.1
C5—C6—H6	117.4	C19—C18—C17	120.2 (2)
C7—C6—H6	117.4	C19—C18—H18	119.9
C9—C7—C8	104.15 (16)	C17—C18—H18	119.9
C9—C7—C6	126.98 (18)	C18—C19—C20	119.5 (2)
C8—C7—C6	128.87 (18)	C18—C19—H19	120.2
N2—C8—C7	111.06 (17)	C20—C19—H19	120.2
N2—C8—C10	118.81 (16)	C19—C20—C21	121.1 (2)
C7—C8—C10	130.13 (17)	C19—C20—H20	119.4
N3—C9—C7	107.91 (17)	C21—C20—H20	119.4
N3—C9—H9	126.0	C16—C21—C20	118.71 (19)
C7—C9—H9	126.0	C16—C21—H21	120.6
C15—C10—C11	118.33 (18)	C20—C21—H21	120.6
C15—C10—C8	119.95 (17)	C2—N1—C3	109.66 (18)
C11—C10—C8	121.69 (17)	C8—N2—N3	105.17 (15)
C12—C11—C10	120.31 (19)	C9—N3—N2	111.72 (16)
C12—C11—H11	119.8	C9—N3—C16	127.74 (16)
C10—C11—H11	119.8	N2—N3—C16	120.41 (15)
C13—C12—C11	120.6 (2)	C4—S1—C2	89.14 (10)
C13—C12—H12	119.7		
			
O1—C1—C2—N1	170.4 (2)	C11—C10—C15—C14	−0.1 (3)
C5—C1—C2—N1	−10.0 (3)	C8—C10—C15—C14	−178.33 (19)
O1—C1—C2—S1	−7.2 (3)	C21—C16—C17—C18	0.4 (3)
C5—C1—C2—S1	172.39 (15)	N3—C16—C17—C18	−178.6 (2)
N1—C3—C4—S1	0.3 (3)	C16—C17—C18—C19	−0.1 (4)
O1—C1—C5—C6	−1.0 (3)	C17—C18—C19—C20	−0.5 (4)
C2—C1—C5—C6	179.36 (19)	C18—C19—C20—C21	0.8 (3)
C1—C5—C6—C7	179.25 (19)	C17—C16—C21—C20	−0.1 (3)
C5—C6—C7—C9	6.8 (3)	N3—C16—C21—C20	178.92 (18)
C5—C6—C7—C8	−173.9 (2)	C19—C20—C21—C16	−0.5 (3)
C9—C7—C8—N2	−0.1 (2)	C1—C2—N1—C3	−177.27 (19)
C6—C7—C8—N2	−179.51 (19)	S1—C2—N1—C3	0.4 (2)
C9—C7—C8—C10	−179.0 (2)	C4—C3—N1—C2	−0.5 (3)
C6—C7—C8—C10	1.5 (3)	C7—C8—N2—N3	0.0 (2)
C8—C7—C9—N3	0.2 (2)	C10—C8—N2—N3	179.03 (16)
C6—C7—C9—N3	179.63 (19)	C7—C9—N3—N2	−0.2 (2)
N2—C8—C10—C15	38.3 (3)	C7—C9—N3—C16	175.49 (18)
C7—C8—C10—C15	−142.9 (2)	C8—N2—N3—C9	0.2 (2)
N2—C8—C10—C11	−139.93 (19)	C8—N2—N3—C16	−175.91 (17)
C7—C8—C10—C11	38.9 (3)	C17—C16—N3—C9	26.8 (3)
C15—C10—C11—C12	0.8 (3)	C21—C16—N3—C9	−152.2 (2)
C8—C10—C11—C12	179.01 (18)	C17—C16—N3—N2	−157.79 (19)
C10—C11—C12—C13	−0.6 (3)	C21—C16—N3—N2	23.2 (3)
C11—C12—C13—C14	−0.2 (3)	C3—C4—S1—C2	−0.04 (19)
C12—C13—C14—C15	0.9 (3)	N1—C2—S1—C4	−0.24 (17)
C13—C14—C15—C10	−0.8 (3)	C1—C2—S1—C4	177.61 (18)

### Hydrogen-bond geometry (Å, º)

**Table:**

*D*—H···*A*	*D*—H	H···*A*	*D*···*A*	*D*—H···*A*
C18—H18···O1^i^	0.94	2.56	3.451 (3)	158
C4—H4···N2^ii^	0.94	2.54	3.473 (3)	172
C13—H13···O1^iii^	0.94	2.41	3.321 (3)	162

Symmetry codes: (i) −*x*+1, *y*−1/2, −*z*+1/2; (ii) *x*−1, *y*, *z*−1; (iii) −*x*+1, −*y*+2, −*z*+1.
